# Extraction Optimization, Preliminary Identification, and Bioactivities in Corn Silk

**DOI:** 10.1155/2023/5685174

**Published:** 2023-02-02

**Authors:** Ping Li, Guangxi Ren, Yifan Sun, Dan Jiang, Chunsheng Liu

**Affiliations:** School of Chinese Materia Medica, Beijing University of Chinese Medicine, Beijing 102488, China

## Abstract

For thousands of years, corn silk has been widely used as an antidiabetic, antioxidant, and antihyperlipidemic and for other effects, but there is a lack of studies that correlate the extracts of flavonoid composition with their biological activities. Thus, the objectives of this study were to optimize the conditions for extracting flavonoids, identify flavonoids, and correlate the flavonoid composition with the biological activities in corn silk. The response surface experiments showed that the highest flavonoid content was predicted at 45.321 min, 57.349°C, 26.089 mL/g, and 71.269%, respectively. The verification experiment results under these optimized conditions showed an ultrasonic time of 45 min, an ultrasonic temperature of 57°C, a liquid-to-material ratio of 26, and an ethanol volume fraction of 70%. No significant differences (the relative error is 4.378%) were observed between the theoretical and experimental TFC values, indicating that the developed models were accurate. Under these optimum extraction conditions, 20 major compounds were identified and quantified by UPLC-LTQ/Orbitrap MS. Furthermore, these optimum ethanol extracts of corn silk are effective against *Bacillus subtilis* and hypoglycemic activity compared with the traditional heating reflux extraction method. Six corn silk components seem to be the main contributors to the inhibitory effect against *Bacillus subtilis* and hyperglycemia activities. These results are useful for the application of corn silk in the food or pharmaceutical industry.

## 1. Introduction

Corn silk, the dried style and stigma of *Zea mays* L., is a well-known traditional Chinese medicine which has been widely used as tea [1, 2], herb medicine [3–5], and food ingredients [6, 7]. Corn silk contains rich flavonoids [5, 8, 9], polysaccharides [10, 11], and terpenoids [12, 13], which possess a variety of biological activities including antioxidant [9], hypoglycemic [6, 14], anticancer [4, 15], and antibacterial properties [16–18]. Among them, the flavonoids in corn silk are closely related to their rich pharmacological activities. Wu et al. showed the antihyperlipidemic activities of total flavonoids from corn silk [19]. Wang et al. proved that the flavonoid-rich extract from corn silk can ameliorate membranous nephropathy activities [20]. After comparing the flavonoid profiles of corn silks, Fougere found only some varieties that showed the efficient inhibitory activity against *Helicoverpa zea*, depending on its flavonoid composition [21]. Although corn silk is rich in flavonoid compounds, very little is known about the extraction, composition of flavonoid compounds, and correlation of flavonoid composition with the biological activities of corn silk.

The choice of an appropriate extraction method is crucial to isolate natural bioactive compounds from plants and materials. Conventional extraction is the most studied and used method to extract compounds. However, it presents a few flaws such as long extraction time and bioactive compound degradation [22]. Therefore, it is of great significance to employ alternative methods. Many research studies have proved that UAE presents extraction yields in a shorter time [23, 24]. Ultrasonic-assisted extraction (UAE) is a simple technique that has the advantages of time-saving, utilisation of a low solvent amount, and high efficiency [25]. It has been widely used to design experiments to optimize extraction process conditions [26–28]. The yield and bioactivities of plant extracts are dependent on the extraction conditions such as type of solvent, liquid-to-solid (L : S) ratio, extraction temperature and time, and particle size. Hence, optimizing an efficient extraction process is very important for a particular variety. The response surface methodology (RSM) is an efficient mathematical and statistical tool that is used to determine and optimize variable experimental conditions to achieve maximum yields with minimum time and less resource consumption [29–31]. The various parameters and their probable interactions can be evaluated efficiently with a mathematical model, reducing the number of experiments.

Ultra-performance liquid chromatography coupled with linear ion trap/Orbitrap mass spectrometry (UPLC-LTQ/Orbitrap MS), a method with high sensitivity and high accuracy, has been widely used to investigate the constituents of medicinal plants such as *Ononis spinosa* L. [32]. Therefore, this study used UAE to extract the total flavonoid from corn silk and use UPLC-LTQ/Orbitrap MS to analyze the chemical constituents of ethanol extracts of corn silk. Unhealthy habits have led to the frequent occurrence of diseases such as bacterial inflammation and hyperglycemia, but drug treatment sometimes may cause adverse reactions [33]. Thus, it is necessary to obtain substances from food extracts to assist or even substitute drug treatments for the treatment of diseases.

To date, ultrasound-assisted extraction with RSM has been used in the extraction of polysaccharides [10], polyphenols [34], and flavonoid [35] compounds from corn silk. However, there is a lack of studies that correlate the extracts of flavonoid compositions with their biological activities. The current study was planned to optimize a given set of extraction conditions and reveal the chemical properties and pharmacological activities of ethanol extracts of corn silk, which might provide a theoretical basis for the further development and utilisation of corn silk resources in the future.

## 2. Materials and Methods

### 2.1. Plant Materials

Corn silk was obtained from Xinxiang, Henan Province, and identified as authentic by Professor Liu Chunsheng of the Beijing University of Chinese Medicine. It was air-dried to constant weight and pulverized, and the powder was passed through a 65-mesh sieve and stored at 4°C.

### 2.2. Standards and Regents

LC/MS grade methanol (MeOH), acetonitrile (ACN), and formic acid (FA) were supplied by Fisher Scientific (Hampton, NJ, USA). Purified water was purchased from Wahaha Group Co., Ltd. (Hangzhou, Zhejiang, China). Rutin standard (B20771, HPLC), sodium phosphate buffer (R2132, pH 6.3), and penicillin-streptomycin solution (S17032, tissue culture grade) were purchased from YuanYe Biology (Shanghai, China). *Bacillus subtilis* (ATCC 6633), *Staphylococcus aureus* (ATCC 6538P), and *Escherichia coli* (BNCC133264) were purchased from BNCC (Beijing, China). The nutrient broth medium (HB0108) was obtained from Hope Bio (Qingdao, Shandong, China). Starch soluble (R014750), *α*-amylase (R00329), and acarbose (R001437) were purchased from Rhawn (Shanghai, China). The remaining reagents were of analytical grade and purchased from local suppliers.

### 2.3. Extraction Methods

#### 2.3.1. Ethanol Reflux Extraction

Ethanol reflux extraction was carried out according to the method described by Wu et al. [19]. In brief, the ethanol extract was refluxed at 80°C for 3 h, taking 1 : 30 as a solid-liquid ratio with 60% ethanol as an extraction solvent.

#### 2.3.2. Ultrasonic-Assisted Extraction

In view of the fact that flavonoids are the main active components of corn silk, we used the total flavonoid content (TFC) as an indicator to optimize the UAE process of corn silk ethanol extracts.

### 2.4. Optimization of the UAE Process

#### 2.4.1. Preliminary Investigations

To find the appropriate range of variables for the extraction process, the ultrasonic power (W), ultrasonic time (min), ultrasonic temperature (°C), liquid-solid ratio (mL/g), and ethanol concentration (%) were tested in a preliminary experiment to investigate the factors and levels of the flavonoid extraction process (Table 1). After soaking in solvent for an hour, the constant extraction conditions in each single-factor test were as follows: 70% ethanol solution 30 mL, an ultrasonic power of 500 W, and an ultrasonic temperature of 60°C for 30 minutes. In the selected conditions, a sample was centrifuged at 4°C and 8000 r/min for 10 minutes, and 1 mL sample was diluted 25∼50 times for subsequent color development.

#### 2.4.2. Experimental Design for RSM

The three-level four-factor Box–Behnken design (BBD) was carried out to optimize the UAE condition (Table 2). A total of 29 experimental runs were used to normalize parameters (Table 3).

The extraction yield of TFC was viewed as the response factor. ANOVA was applied to estimate the influence of each factor. The *P* value of the regression model and the regression coefficient were used to evaluate the scientific model. Three-dimensional pictures and two-dimensional pictures were used to demonstrate the influence of the interaction of the two factors on extraction.

### 2.5. Determination of TFC

TFC was determined based on the method reported by Sabiu et al. [36] with a slight modification. Briefly, 1 mL of extracted samples was dissolved in 14 mL of 70% ethanol and 0.7 mL of 5% NaNO_2_ solution. After 5 min, 0.7 mL of AlCl_3_ (10% w/v) was added to the solution. The mixture was allowed to stand for 5 min, and 5 mL of NaOH solution (1 mol/L) was added. Finally, we added 70% ethanol to dilute it to 25 mL, and it was reacted and mixed well and incubated at room temperature for 15 min. Then, the absorbance was measured at 510 nm. Rutin was used to make the standard calibration curve for TFC. It (10 mg) was dissolved in 50 mL of 70% ethanol, after which serial dilutions were prepared with ethanol (0.016–0.08 mg/mL). The TFC calculation equation was as follows: *Y* = (*C* × *V* × *T*/1000) × 100% (*Y*: TFC, *C*: absorbance content, *V*: solution volume, and *T*: dilution times).

### 2.6. *In Vitro* Activities

#### 2.6.1. Antibacterial Assay

Three laboratory strains (*Bacillus subtilis*, *Staphylococcus aureus*, and *Escherichia coli*) from our laboratory were used for the antibacterial assay. The Oxford cup method was used to determine the antibacterial activity of the corn silk ethanol extract. Before the assay was performed, bacterial cultures were refreshed in media and kept at 37°C. The sample was processed with the optimized process after fine-tuning and refluxing of ethanol. The extracted corn silk sample solution was freeze-dried to a constant weight, after which serial dilutions were prepared with 70% ethanol (80–240 mg/mL). The Petri dishes were cultured at 37°C for 16 h. The size of the inhibition zones was observed and recorded. Normal saline was used as a negative control, and the penicillin-streptomycin solution (penicillin G, sodium salt: 10k U/mL; streptomycin sulfate: 10 mg/mL) was used as a positive control. The minimum inhibitory concentration (MIC) was determined by the double dilution method [37, 38]. The extracted corn silk sample solution was freeze-dried to a constant weight and then diluted to a range of concentrations (6.25–200 mg/mL) with 70% ethanol. The inoculated plates were incubated at 37°C for 16 h. The MIC was defined as the lowest concentration that prevented the color from changing. MIC determination was performed on different 3 days, and each day, it was performed in triplicate.

#### 2.6.2. Anti-*α*-Amylase Assay

It has been reported that the corn silk ethanol extract has *α*-amylase inhibitory activity [36]. Herein, the effect of the reaction time and extract concentration on the inhibition rate was investigated by 3,5-dinitrosalicylic acid colorimetry [39]. We added 0.5 mL *α*-amylase to 0.5 mL of different dilutions (0.5 mg/mL, 1.0 mg/mL, and 1.5 mg/mL). After that, at 37°C, water was bathed for 20 min, 30 min, and 40 min, and we added a 1% starch solution of 1 mL. At 37°C, water was bathed for 15 min. Then, we added 1 mL DNS for color development, and boiling water was bathed for 5 min and cooled to 25°C; finally, we added 10 mL deionized water. The absorbance was measured at 540 nm. Each sample was tested in triplicate. Triplicates of the negative control (deionized water) and the positive control (acarbose aqueous solution) were also measured. The following formula was used to calculate the *α*-amylase inhibitory activity: *Y* = (1 − ((X1 − X2)/X3))×100% (X1: *α*-amylase solution, sample, starch solution, and DNS developer, X2: starch solution, sample, and DNS developer, and X3: *α*-amylase solution, starch solution, and DNS developer).

### 2.7. Qualitative Analysis

The UPLC system used a Thermo Scientific Dionex UltiMate 3000 UHPLC Plus Focused ultrahigh-liquid chromatography system equipped with a binary gradient pump, autosampler, column thermostat, and DAD detector. Chromatographic separation was conducted on an ACQUITY UPLC BEH C18 column (2.1 mm × 100 mm, 1.7 *μ*m; Waters corp., Milford, MA, USA) at a temperature of 40°C and an absorption wavelength of 254 nm. The mobile phase consisted of 0.1% FA water (A) and ACN (B) with linear gradient elution at a flow rate of 0.3 mL/min. The sample injection volume was 2 *μ*L. The gradient elution program was set as follows, as shown in Table 4.

The LTQ-Orbitrap XL linear ion trap-tandem electrostatic field Orbitrap mass spectrometer was equipped with a thermal spray ion source (HESI), and Xcalibur 2.1 ChemStation was operated in a negative ion detection mode. The ion source temperature was 350°C, the ionization source voltage was 4 kV, the capillary voltage was 35 V, the tube lens voltage was 110 V, and the sheath gas and auxiliary gas were both high-purity nitrogen (purity > 99.99%). The sheath gas flow rate was 40 arb, and the auxiliary gas flow rate was 20 arb. The data adopted the Fourier transform high-resolution full scan mode (TF, full scan, resolution 30000) for data-dependent acquisition of ddMS^2^ and ddMS^3^.

### 2.8. Correlation Analysis of Corn Silk Components with Their Biological Activities

#### 2.8.1. Screening of Related Targets

We collected the component targets using the TCMSP (http://lsp.nwu.edu.cn/tcmsp.php) database. After screening, the UniProt protein database (https://www.uniprot.org) was unified to standardize the protein targets on which compounds act to standardize protein target information. Mining relevant targets was carried out by setting “Bacillus subtilis,” “hyperglycemia,” and “hyperglycemia” “as key words” in the DisGeNET (https://www.disgenet.org), TTD database (http://bidd.nus.edu.sg/group/cjttd), GeneCards database (https://www.genecards.org), DRUGBANK database (https://www.drugbank.ca), and OMIM database (http://www.omim.org). In the GeneCards and DisGeNET databases, the target with a score greater than or equal to the median was set as the potential target. The results of the five databases were combined, duplicate values were deleted, and the result was the target related to *Bacillus subtilis* or hyperglycemia.

#### 2.8.2. Construction of the PPI Network

Venny2.1 (https://bioinfogp.cnb.csic.es/tools/venny/) was used to analyze the relationship between the identified components and *Bacillus subtilis* or hyperglycemia targets. The intersection was found, and a Venn diagram was drawn. The intersection target was submitted to the STRING database (https://string-db.org) to construct a protein interaction (PPI) network model. 1st and 2nd shells were set as no more than 5 interactors.

### 2.9. Statistical Analysis

All experiments were performed in triplicates, and average values with standard deviation errors were reported. Statistical analysis was performed using Design-Expert ver. 12 (Stat-ease INC., Minneapolis, MN, USA) and IBM SPSS ver. 23 statistic software (IBM, Armonk, NY, USA). The analysis of variance was applied for assessing statistically significant differences between the samples (*P* < 0.05) using ANOVA. The chemical analyses were monitored and performed with Xcalibur 4.0 software (Thermo Fisher Scientific, MA, USA). GraphPad Prism ver.8.3.0 (GraphPad Software INC., San Diego, CA, USA) and Adobe Illustrator ver. 2020 (Adobe INC., San Jose, CA, USA) were applied to mapping analyses.

## 3. Results and Discussion

### 3.1. TFC of Ethanol Reflux Extraction

We conducted three experiments in parallel, and the average yield was 1.152% ± 0.09%, which was quite different from the yield obtained by ultrasonic extraction. It can be seen that in this study, ultrasonic extraction showed obvious advantages in high efficiency.

### 3.2. TFC of the UAE Process

**Figure 1:**
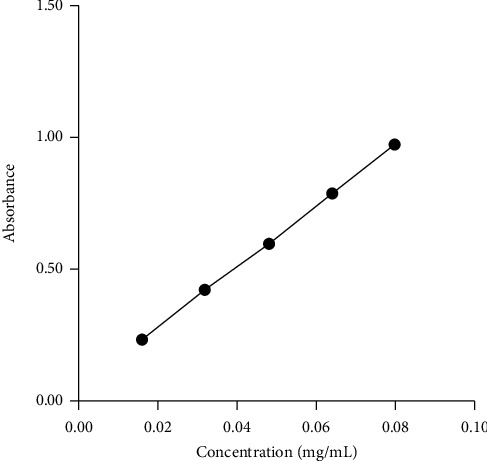
The standard curve of rutin in the range of 0.016 mg/ml–0.08 mg/ml.

**Figure 2:**
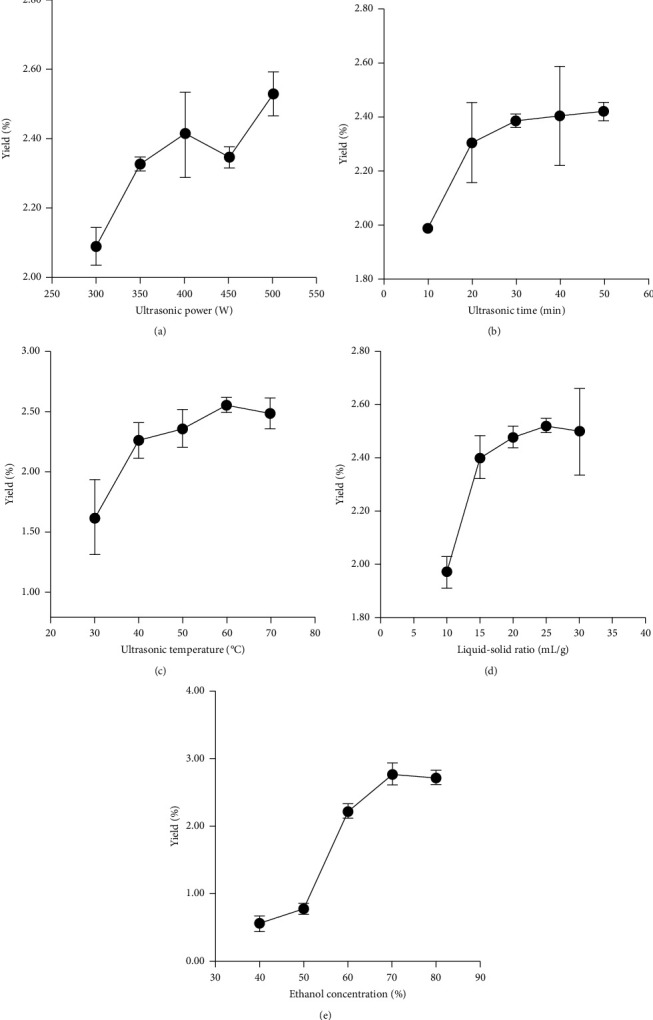
Variation of the total flavonoid yield of corn silk with different single factors. (a) Ultrasonic power, (b) ultrasonic time, (c) ultrasonic temperature, (d) liquid-solid ratio, and (e) ethanol concentration.

The rutin standard curve equation was *y* = 11.565*x* + 0.0497, *R*^2^ = 0.9998, which illustrated that there was a good linear relationship between the mass concentration and the absorbance value in the range of 0.016 mg/mL–0.08 mg/mL (Figure 1). The influence of different single factors on TFC ultrasonic extraction is shown in Figure 2. According to the experimental results, the factors and levels of RSM are determined, as shown in Table 2.

#### 3.2.1. Results of RSM

The fitted multiple regression equation is *Y* = 2.41 + 0.0457 ∗ A − 0.13 ∗ B − 0.0502 ∗ C + 0.0883 ∗ *D* + 0.0298 ∗ AB − 0.0247 ∗ AC − 0.0605 ∗ AD − 0.0312 ∗ BC + 0.03 ∗ BD + 0.015 ∗ CD − 0.1906A^2^ − 0.2469B^2^ − 0.1346C^2^ − 0.3151D^2^.

The regression model analysis of variance is shown in Table 5. *R*^2^ = 0.9656; *R*_Adj_^2^ = 0.9312; the CV value was 2.85%. It can be seen in Table 5 that the model had a value of *P* < 0.001, indicating that the regression model was extremely significant; the lack of the fit item was not significant (*P* > 0.05), implying that the quadratic regression model fitted well with the actual situation. The primary and secondary orders of factors affecting the yield of corn silk flavonoids were B > D > C > A.

#### 3.2.2. Response Surface Analysis

**Figure 3:**
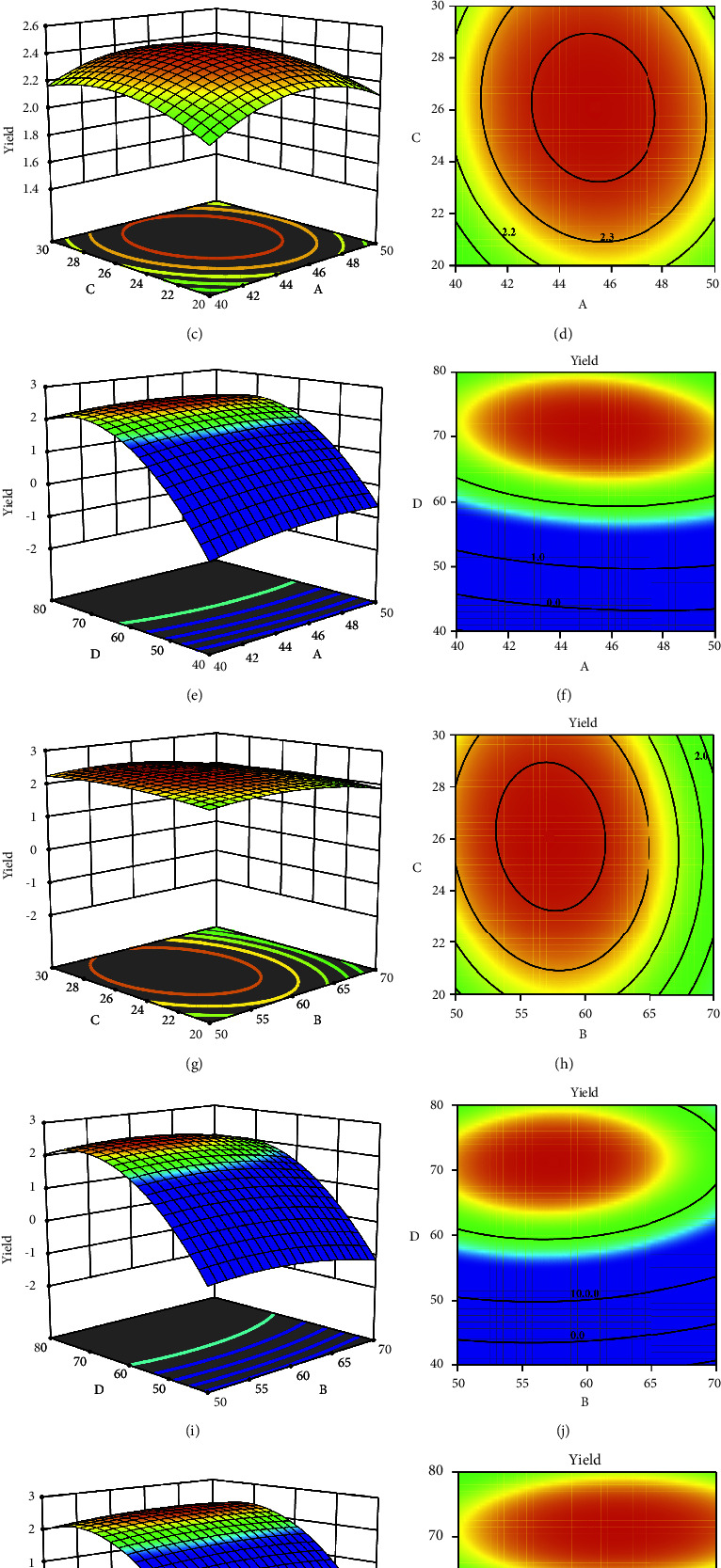
The effect of the interaction of two factors on the yield of corn silk flavonoids. (a) The 3-dimensional model of the interaction of ultrasonic time and ultrasonic temperature on the yield of corn silk flavonoids. (b) The 2-dimensional model of the interaction of ultrasonic time and ultrasonic temperature on the yield of corn silk flavonoids. (c) The 3-dimensional model of the interaction of ultrasonic time and liquid-solid ratio on the yield of corn silk flavonoids. (d) The 2-dimensional model of the interaction of ultrasonic time and liquid-solid ratio on the yield of corn silk flavonoids. (e) The 3-dimensional model of the interaction of ultrasonic time and ethanol concentration on the yield of corn silk flavonoids. (f) The 2-dimensional model of the interaction of ultrasonic time and ethanol concentration on the yield of corn silk flavonoids. (g) The 3-dimensional model of the interaction of ultrasonic temperature and liquid-solid ratio on the yield of corn silk flavonoids. (h) The 2-dimensional model of the interaction of ultrasonic temperature and liquid-solid ratio on the yield of corn silk flavonoids. (i) The 3-dimensional model of the interaction of ultrasonic temperature and liquid-solid ratio on the yield of corn silk flavonoids. (j) The 2-dimensional model of the interaction of ultrasonic temperature and liquid-solid ratio on the yield of corn silk flavonoids. (k) The 3-dimensional model of the interaction of liquid-solid ratio and ethanol concentration on the yield of corn silk flavonoids. (l) The 2-dimensional model of the interaction of liquid-solid ratio and ethanol concentration on the yield of corn silk flavonoids. Meaning of coordinate axis: (A) ultrasonic time (min), (B) ultrasonic temperature (°C), (C) liquid-solid ratio (mL/g), and (D) ethanol concentration (%).

The response surface-contour map is shown in Figure 3. It can be seen in the figure that the ultrasonic temperature surface was steepest, indicating that it had the greatest impact on the response value; the ultrasonic time response surface had the gentlest slope, and its impact on the response value was least. The optimized extraction process parameters obtained by response surface analysis were as follows: the ultrasonic time was 45.321 min, the ultrasonic temperature was 57.349°C, the liquid-to-material ratio was 26.089, and the ethanol volume fraction was 71.269%. Under these conditions, the theoretical prediction value of the flavonoid yield was 2.444%.

#### 3.2.3. Verification Test Results

In view of the limitations of the actual operation, the flavonoid extraction process conditions were adjusted to an ultrasonic time of 45 min, an ultrasonic temperature of 57°C, a liquid-to-material ratio of 26, and an ethanol volume fraction of 70%. Under these conditions, three experiments were performed to verify the optimized conditions. The average yield was 2.337% ± 0.118%, and the relative error between the theoretical prediction value was 4.378%, which was less than 5%, indicating that the extraction process parameters obtained by applying RSM optimization were reliable.

Previous researchers have designed RSM to determine the optimal extraction conditions of total flavonoids in corn silk [40]. The results are quite different from those of this experiment, which may be due to the fact that the ultrasonic temperature or ultrasonic power was not considered for the single-factor test.

### 3.3. *In Vitro* Activities

#### 3.3.1. Antibacterial Activity

**Figure 4:**
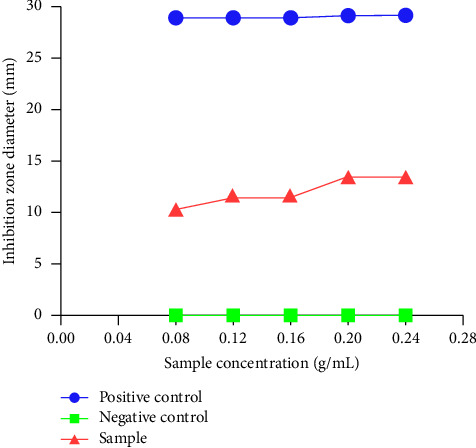
Different concentrations of corn silk ethanol extracts had different inhibitory effects on *Bacillus subtilis*. The criteria for antibacterial effects are as follows: diameter >20 mm means extremely effective, 15∼20 mm means highly effective, 10∼15 mm means moderately effective, 7∼9 mm means slightly effective, and absence of inhibition zones means ineffective.

The corn silk of RSM-optimized extraction exhibited an inhibitory effect against *B. subtilis* (Figure 4). However, there were no antibacterial activities against *S. aureus* and *E. coli*. As is shown in Figure 4, these data for the inhibition of bacterial activity showed dose-dependent relationships for all of these extracts: the higher the concentration of the applied extract, the higher the inhibition of bacterial activity. The higher the concentration of the extract, the better the inhibitory effect with MIC values of 25 mg/mL. Moreover, the corn silk obtained by ethanol reflux had no inhibitory effect on three bacterial strains. It was speculated that ultrasonic extraction had the ability to extract more antibacterial active substances from corn silk. At present, the application of corn silk along with synthesis exhibited antibacterial activity toward Gram-positive and Gram-negative bacteria, as a clean and cheap agricultural waste [41].

#### 3.3.2. Anti-*α*-Amylase Activity

In the *α*-amylase inhibitory activity assay, the results show that the ethanol extract of corn silk has inhibitory effects (Tables 6–8). The concentration of the sample and the reaction time affected the inhibitory effect. The results indicated that the corn silk ethanol extract demonstrated the activity of inhibiting *α*-amylase, and the inhibitory effect increases with an increase in concentration at 0.5∼1.5 mg/mL. Under the test conditions, the inhibition effect is best when the sample concentration is 1.5 mg/mL and the reaction time is 30 minutes. When the reaction time was 40 minutes, the inhibition rate was very low, and we speculated that this may be due to this inhibition being reversible.

However, considerable differences across these extracts were seen for different extraction methods. The inhibition rate of ethanol reflux was significantly lower than that of ultrasonic extraction (Tables 6 and 7). It can be seen that the ultrasonic extraction method was more effective in extracting components that inhibit the activity of *α*-amylase. It can thus be concluded that corn silk is a promising inhibitor of *α*-amylase and can therefore be considered as antidiabetic potential.

The conclusion that corn silk extracts can be considered as antidiabetic agents is here partly based on previous studies that have shown anti-*α*-amylase and anti-*α*-glucosidase activities for polysaccharides [6] and polyphenols [5]. However, the data in the present study demonstrate that these corn silk extracts that contain quercetin have a direct impact on the *α*-amylase activity and pointed out the fact that other substances present in these extracts may have important impacts on the overall anti-*α*-amylase activity, which was not shown by other studies. These data reinforce the possibility of using corn silk extracts in the human diet and could be further developed as a cheap and plant-derived agent in the management of diabetes.

### 3.4. Qualitative Analysis

**Figure 5:**
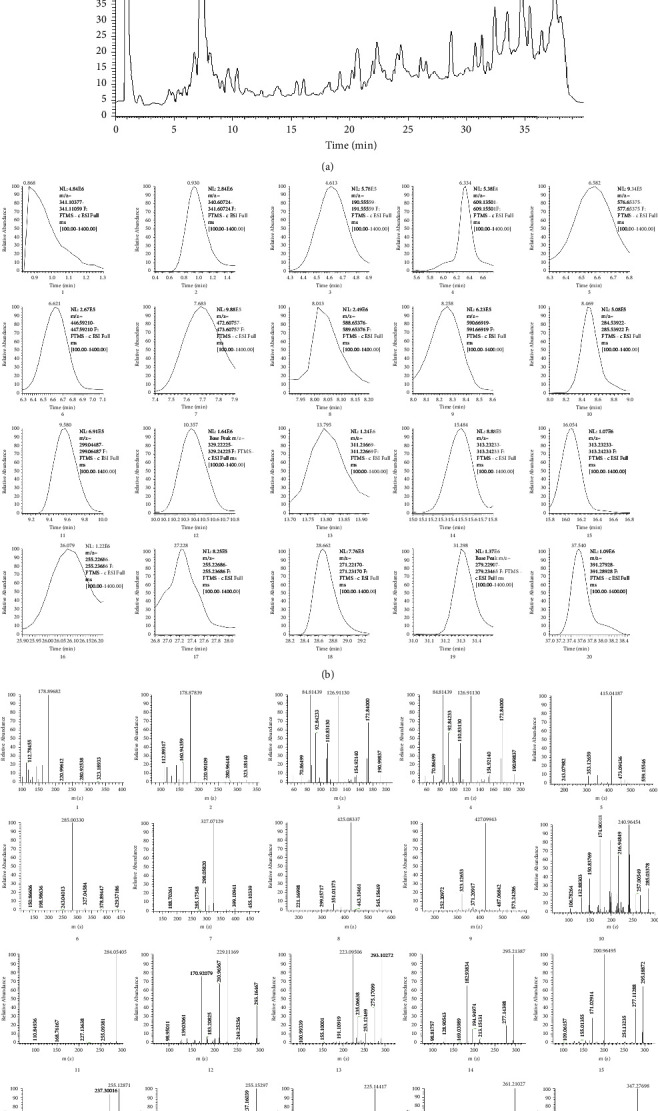
The results of qualitative analysis. (a) TIC of the ethanol extract of corn silk in a negative ion mode. (b) The chromatograms of 20 compounds. (c) The secondary fragment ion patterns of 20 compounds.

**Figure 6:**
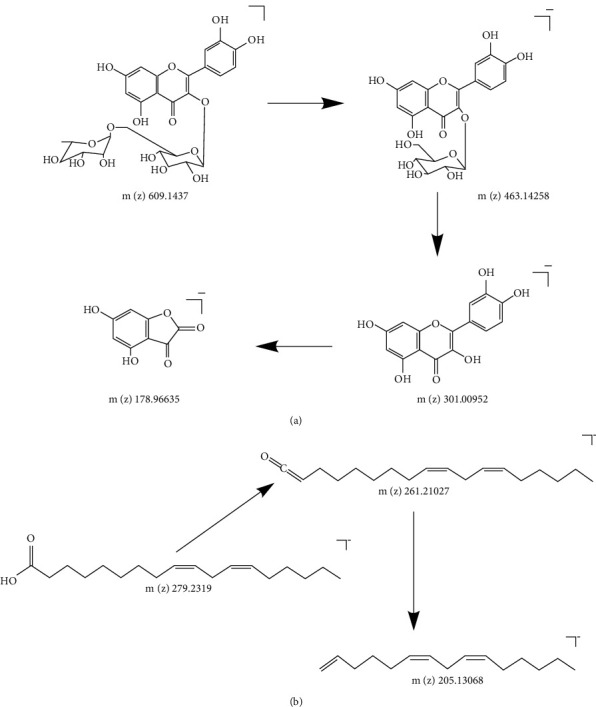
Typical fragmentation pathways of two main chemical components. (a) The fragmentation pathway of rutin. (b) The fragmentation pathway of linoleic acid.

The determination of total flavonoids and the results of *in vitro* antibacterial tests show that ultrasonic extraction can obtain more active ingredients than reflux extraction. Therefore, we conducted a qualitative analysis of the solution obtained by ultrasonic extraction. A total of 20 compounds (Table S1) were identified, which include two carbohydrates, one organic acid, eight flavonoids, seven fatty acids, one ester, and one steroid. We confirmed the components by combining mass spectrometry fragmentation law, database data (https://www.chemspider.com), literature data, MS, MS2, retention time and assumed formula [42–46]. Total ion chromatography (TIC) is shown in Figure 5(a), the chromatograms of 20 compounds are shown in Figure 5(b), and the secondary fragment ion patterns of 20 compounds are shown in Figure 5(c). According to the results, flavonoids and fatty acids are the main components. We took rutin as an example to analyze the ionization process of flavonoids (Figure 6(a)), and we took linoleic acid as an example to analyze the ionization process of fatty acids (Figure 6(b)).

### 3.5. Correlation Analysis

#### 3.5.1. Related Targets

Through the TCMSP database and UniProt protein databases, 7 targets of the identified components were found, as shown in Table S2. The seven compounds are sucrose (carbohydrate), D-(-)-quinic acid (organic acids), rutin (flavonoids), luteolin (flavonoids), hispidulin (flavonoids), palmitic acid (fatty acids), and deoxycholic acid (steroids). Among the seven compounds, sucrose is related to *B. subtilis* and hyperglycemia activity because it can promote the growth of *B. subtilis* as a carbon source [47] and induce hyperglycemia [48]. While all the other six components have antibacterial activity and can ameliorate hyperglycemia activity.

A total of 115 targets were found, among which 93 targets were found from three flavonoids, rutin, luteolin, and hispidulin, which implies that the flavonoids in the extract are the main active ingredients. After deleting duplicate values, there are 90 targets related to 6 components. 196 targets are related to *B. subtilis* obtained from the 5 databases, and 1182 targets are related to hyperglycemia.

#### 3.5.2. PPI Network

**Figure 7:**
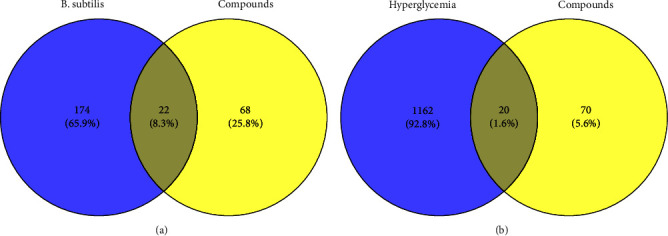
Targets related to 6 components with *Bacillus subtilis* and hyperglycemia. (a) 22 common targets related to 6 components and *Bacillus subtilis*. (b) 20 common targets related to 6 components and hyperglycemia.

**Figure 8:**
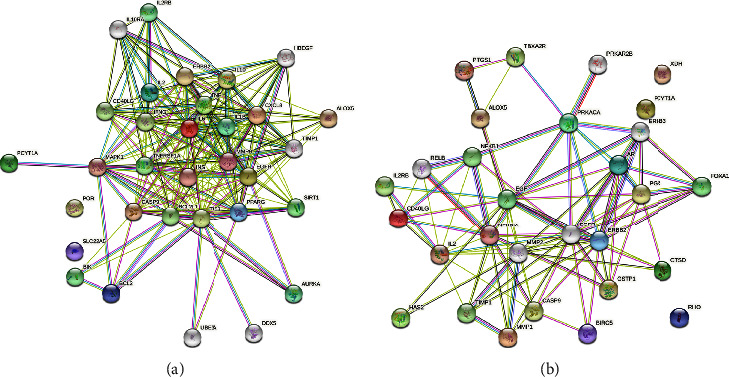
PPI network of related targets. (a) PPI of common targets related to 6 components and *Bacillus subtilis.* (b) PPI network of common targets related to 6 components and hyperglycemia.

There were 22 common targets related to 6 components and *B. subtilis* (Figure 7(a)), and 20 targets were related to hyperglycemia (Figure 7(b)). We submitted the common target to STRING to obtain the PPI network (Figure 8). In summary, the 6 corn silk components were related to *B. subtilis* and hyperglycemia-related targets, and their common targets interacted with each other, which provided a theoretical basis for studying the pharmacological effects of corn silk.

## 4. Discussion

Conventional extraction techniques (maceration and ethanol reflux) require high consumption of available resources and thus are ineffective and expensive. In this study, the response surface methodology was used to optimize the ultrasonic extraction of total flavonoids from corn silk, which improved yield and had a low processing time compared with ethanol reflux extraction. Previous studies reported the successful extraction of flavonoids using ultrasonic waves from other plants, such as *Ocimum basilicum* [49] and immature *Citrus unshiu* pomace [50]. UAE has a greater effect on the content of extractive substances than other conventional extraction methods [51]. The UAE extraction procedure has the effect of cavitation and the better degradation of plant tissue so that the extraction of desirable bioactive compounds from the plant material can be easier [52]. Because of these reasons, UAE can be considered the technique of choice for extraction of total flavonoid compounds from corn silk.

Many natural products contain a variety of bioactive compounds. Natural compounds play an important role in our daily diet and are part of pharmaceutical and cosmetic industries as well. Therefore, it is crucial to find antibacterial active ingredients from natural compounds. We have proved the antibacterial ability of corn silk and screened related compounds preliminarily, which can provide reference for the development of antibacterial drugs using corn silk in the future. In addition, we have proved that corn silk can be considered an antihyperglycemia candidate. At present, corn silk has been widely used as tea [1, 2] and food ingredients [6, 7]. That is to say, to relieve the effect of the symptoms of hyperglycemia, the reasonable consumption of corn silk as a functional food is feasible.

Nature is regarded as the best source for medicines as a variety of natural compounds exist with promising medicinal values. Here, we identified 24 components from corn silk extracts and found seven components related to *B. subtilis* and hyperglycemia activity. Among them, sucrose is related to *B. subtilis* and hyperglycemia activity because it may contribute to the growth of *B. subtilis* [47] and induce hyperglycemia [48]. While all the other six components have antibacterial activity and can ameliorate hyperglycemia activity. Studies have shown that quinic acid can inhibit the growth of a variety of bacteria [53, 54], and it is a competitive inhibitor of *α*-amylase [55]. Chitosan/silver (CS/Ag) nanocomposite using rutin has antibacterial activity against *B. subtilis* [22]; in diabetic rat models, rutin can significantly reduce fasting blood sugar [56]. Luteolin and hispidulin have antibacterial activity against *B. subtilis* as well [57, 58], and it is reported that they can ameliorate hyperglycemia [59, 60]. Palmitic acid has antibacterial activity [61], and a compressed mixture of insulin and palmitic acid can sustain a reduction in hyperglycemia in rodents [62]. Additionally, research shows that deoxycholic acid-triazole conjugates have good inhibitory effects against *B. subtilis* [63]; deoxycholic acid is involved in improving glucose metabolism to ameliorate hyperglycemia [64]. In short, we have found 6 possible components of corn silk with antibacterial and hyperglycemia activities. This finding will lay a theoretical foundation for expanding the application of corn silk.

In summary, optimization extraction, preliminary characterization, and antibacterial and anti-*α*-amylase activities *in vitro* of corn silk flavonoids were carried out. The optimum extraction conditions of the total flavonoid content were determined using the single-factor experiments and the orthogonal array test. Under optimum reaction conditions, UAE was an effective method to extract flavonoids from corn silk compared with ethanol reflux extraction. UAE provides a higher extraction yield and can obviously reduce the extraction time. Furthermore, the ethanol extract of corn silk is rich in a variety of chemical ingredients and has an inhibitory effect against *B. subtilis* and *α*-amylase. Six corn silk components seem to be the main contributors to the inhibitory effect against *B. subtilis* and hyperglycemia activities. These findings are important for further research on the chemical composition and biological activity of the corn silk ethanol extract, which is conducive to the rational use of corn silk resources. Our results suggested that corn silk could be a proper candidate for bacteriostatic and hypoglycemic agents due to the presence of bioactive compounds. In the future, we recommend isolating and purifying active compounds and performing more *in vitro* and *in vivo* studies for their potential pharmacological applications.

## Figures and Tables

**Table 1:** The factors and levels of the single-factor experiment of the flavonoid extraction process.

Variables	Levels
Ultrasonic power (W)	300, 350, 400, 450, 500
Ultrasonic time (min)	10, 20, 30, 40, 50
Ultrasonic temperature (°C)	40, 50, 60, 70, 80
Liquid-solid ratio (mL/g)	10, 15, 20, 25, 30
Ethanol concentration (%)	40, 50, 60, 70, 80

**Table 2:** The factors and levels of the RSM experiment of the flavonoid extraction process.

Variables	Code	Level		
		−1	0	1
Ultrasonic time (min)	A	40	45	50
Ultrasonic temperature (°C)	B	50	60	70
Liquid-solid ratio (mL/g)	C	20	25	30
Ethanol concentration (%)	D	60	70	80

**Table 3:** The experimental design and results of 29 runs for the RSM experiment of the flavonoid extraction process.

No.	A	B	C	D	Yield (%)
1	45	70	20	70	1.931
2	45	70	25	80	1.823
3	45	60	25	70	2.479
4	50	60	30	70	2.136
5	45	50	20	70	2.136
6	45	50	25	80	2.004
7	40	60	30	70	2.076
8	45	60	25	70	2.457
9	45	50	30	70	2.280
10	45	70	25	60	1.569
11	45	50	25	60	1.870
12	45	60	20	80	1.980
13	40	70	25	70	1.799
14	50	50	25	70	2.082
15	45	60	25	70	2.387
16	45	70	30	70	1.950
17	40	60	25	80	2.008
18	40	60	20	70	1.921
19	45	60	25	70	2.360
20	50	70	25	70	1.870
21	45	60	30	60	1.906
22	50	60	25	80	2.040
23	45	60	20	60	1.822
24	40	50	25	70	2.130
25	45	60	30	80	2.124
26	40	60	25	60	1.739
27	45	60	25	70	2.387
28	50	60	20	70	2.080
29	50	60	25	60	2.013

**Table 4:** Gradient elution program of the qualitative analysis.

Retention (min)	%A	%B
0	95.0	5.0
1	95.0	5.0
8	60.0	40.0
15	50.0	50.0
27	30.0	70.0
35	10.0	90.0
36	10.0	90.0
37	95.0	5.0
40	95.0	5.0

**Table 5:** The regression model analysis of variance.

Source	Sum of squares	d*f*	Mean square	*F* value	*P* value	Significance
Model	1.34	14	0.0956	28.08	<0.0001	^*∗∗∗*^
A	0.025	1	0.025	7.35	0.0169	^*∗*^
B	0.2028	1	0.2028	59.57	<0.0001	^*∗∗∗*^
C	0.0302	1	0.0302	8.87	0.01	^*∗*^
D	0.0936	1	0.0936	27.5	0.0001	^*∗∗*^
AB	0.0035	1	0.0035	1.04	0.3251	
AC	0.0025	1	0.0025	0.7197	0.4105	
AD	0.0146	1	0.0146	4.3	0.057	
BC	0.0039	1	0.0039	1.15	0.3022	
BD	0.0036	1	0.0036	1.06	0.3212	
CD	0.0009	1	0.0009	0.2644	0.6152	
A^2^	0.2357	1	0.2357	69.24	<0.0001	^*∗∗∗*^
B^2^	0.3953	1	0.3953	116.12	<0.0001	^*∗∗∗*^
C^2^	0.1176	1	0.1176	34.53	<0.0001	^*∗∗∗*^
D^2^	0.6441	1	0.6441	189.21	<0.0001	^*∗∗∗*^
Residual	0.0477	14	0.0034			
Lack of fit	0.0372	10	0.0037	1.42	0.3918	
Pure error	0.0104	4	0.0026			
Cor total	1.39	28				

*Note*. ^*∗*^*P* < 0.05, the difference is significant; ^*∗∗*^*P* < 0.01, the difference is highly significant; ^*∗∗∗*^*P* < 0.001, the difference is extremely significant.

**Table 6:** The inhibitory effect of the corn silk ethanol extract on *α*-amylase changed with the change in the sample concentration and reaction time (UAE).

Corn silk ethanol extract concentration (mg/mL)	Inhibition rate (%)		
	20 min	30 min	40 min
0.5	29.11 ± 0.03	31.15 ± 0.23	22.67 ± 0.11
1.0	36.81 ± 0.11	37.21 ± 0.15	29.28 ± 0.31
1.5	42.17 ± 0.02	48.51 ± 0.16	36.92 ± 0.07

**Table 7:** The inhibitory effect of the corn silk ethanol extract on *α*-amylase changed with the change in the sample concentration and reaction time (ethanol reflux).

Corn silk ethanol extract concentration (mg/mL)	Inhibition rate (%)		
	20 min	30 min	40 min
0.5	19.32 ± 0.03	21.43 ± 0.33	17.52 ± 0.21
1.0	27.71 ± 0.53	30.13 ± 0.32	23.28 ± 0.34
1.5	32.17 ± 0.14	38.76 ± 0.23	31.92 ± 0.33

**Table 8:** The inhibitory effect of acarbose aqueous solution on *α*-amylase changed with the change in the sample concentration and reaction time.

Acarbose aqueous solution concentration (mg/mL)	Inhibition rate (%)		
	20 min	30 min	40 min
0.5	20.65 ± 0.31	31.69 ± 0.06	12.94 ± 0.03
1.0	34.23 ± 0.02	36.43 ± 0.19	13.47 ± 0.12
1.5	37.64 ± 0.18	45.07 ± 0.04	14.06 ± 0.18

## Data Availability

No publicly archived datasets were used to support the findings of this study.

## Supplementary Materials

Supplementary Materials
*Table S1*. Characterization of compounds in the ethanol extract of corn silk by UPLC-Q-Orbitrap (negative ion mode). *Table S2.* Targets related to 7 identified corn silk components.
