# Supplementary material for: Extraction Optimization, Preliminary Identification, and Bioactivities in Corn Silk
Source: Evid Based Complement Alternat Med. 2023 Feb 2;2023:5685174. doi: 10.1155/2023/5685174 (PMC9911244; doi:10.1155/2023/5685174)
Supplement: Supplementary Materials — Table S1. Characterization of compounds in the ethanol extract of corn silk by UPLC-Q-Orbitrap (negative ion mode). Table S2. Targets related to 7 identified corn silk components. [file 5685174.f1.doc]

**Table S1.** Characterization of compounds in ethanol extract of corn silk by UPLC-Q-Orbitrap (negative ion mode).

| **No.** | **T_R_(min)** | **Formula** | **Obser. Mass (m/z)** | **Ion mode** | **Theo. Mass  (m/z)** | **Error  (ppm)** | **MS/MS fragments  (m/z)** | **Structure** | **Identification** | **Type** |
| --- | --- | --- | --- | --- | --- | --- | --- | --- | --- | --- |
| 1 | 0.868 | C12 H22 O11 | 341.1072 | [M-H]^-^ | 341.1078 | -1.935 | 178.89682,160.87573,142.97867,112.78455,118.84392 | 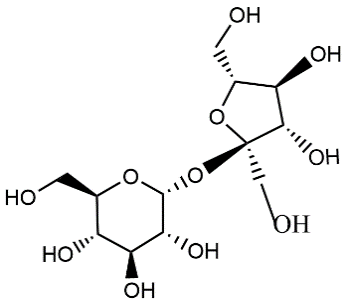 | Sucrose | carbohydrate |
| 2 | 0.930 | C12 H22 O11 | 341.1072 | [M-H]^-^ | 341.1078 | -1.759 | 323.18140, 297.03308, 178.87839, 160.94359, 118.91438 | 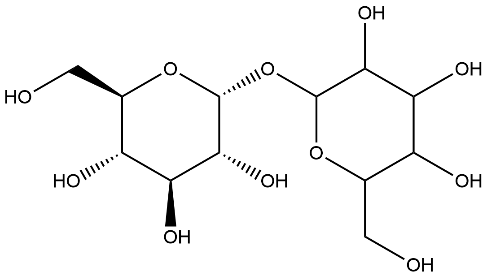 | α,α-Trehalose | carbohydrate |
| 3 | 4.613 | C7 H12 O6 | 191.0556 | [M-H]^-^ | 191.0550 | 3.036 | 172.84000, 126.91130, 110.83130, 92.84233 | 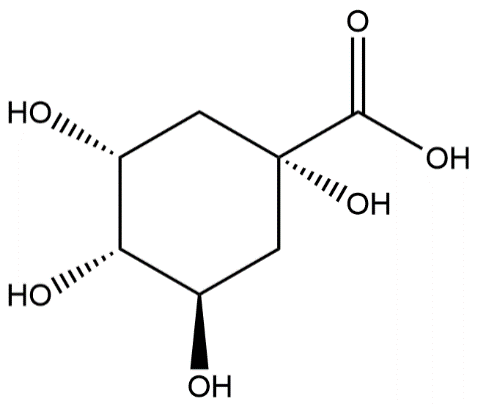 | D-(-)-Quinic acid | Organic acids |
| 4 | 6.334 | C27 H30 O16 | 609.1437 | [M-H]^-^ | 609.1450 | -2.183 | 301.00952,463.14258,178.96635 | 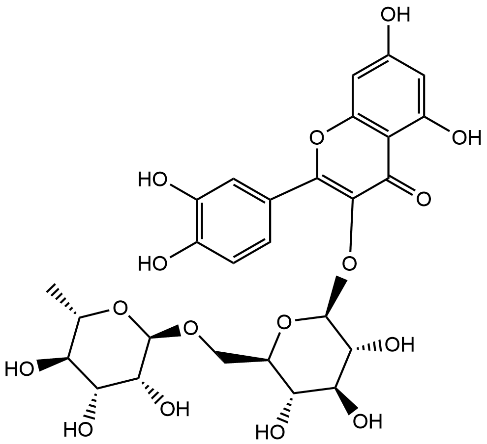 | Rutin | Flavonoids |
| 5 | 6.582 | C27 H30 O14 | 577.1538 | [M-H]^-^ | 577.1552 | -2.478 | 559.15546, 473.09436, 415.04178, 353.12659, 243.07982 | 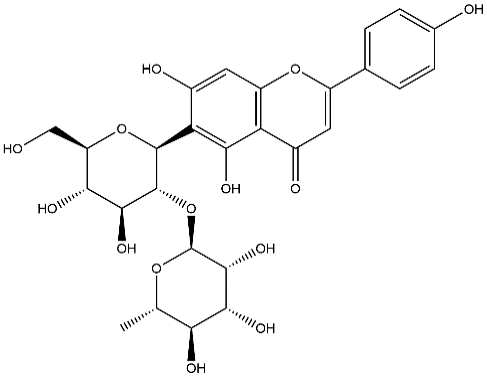 | (1S)-1,5-Anhydro-2-O-(6-deoxy-α-L-mannopyranosyl)-1-[5,7-dihydroxy-2-(4-hydroxyphenyl)-4-oxo-4H-chromen-6-yl]-D-glucitol | Flavonoids |
| 6 | 6.621 | C21 H20 O11 | 447.0921 | [M-H]^-^ | 447.0922 | -0.201 | 429.37186, 378.89447, 327.04584, 285.00330, 243.04013, 198.98636 | 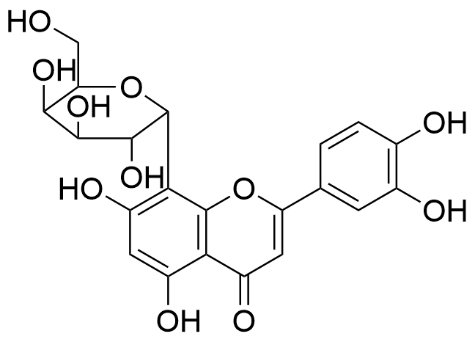 | 1,5-Anhydro-1-[2-(3,4-dihydroxyphenyl)-5,7-dihydroxy-4-oxo-4H-chromen-8-yl]-D-galactitol | Flavonoids |
| 7 | 7.683 | C23 H22 O11 | 473.1076 | [M-H]^-^ | 473.1078 | -0.571 | 429.26340, 399.10941, 369.00375, 327.07129 | 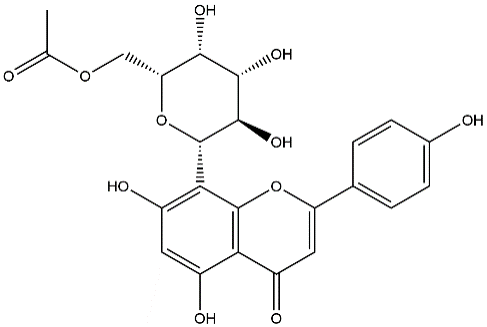 | Apigenin-8-C- (6"-acetylgalactoside) | Flavonoids |
| 8 | 8.013 | C28 H30 O14 | 589.1538 | [M-H]^-^ | 589.1552 | -2.410 | 545.15649, 443.10461, 425.08337, 381.08362, 323.05139 | 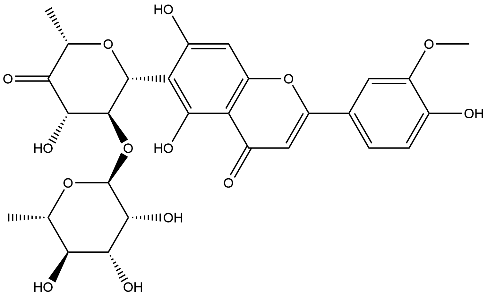 | 3'-O-Methylmaysin | Flavonoids |
| 9 | 8.258 | C28 H32 O14 | 591.1692 | [M-H]^-^ | 591.1708 | -2.774 | 573.24286, 487.06842, 427.09943, 371.20917, 323.12653,196.91023 | 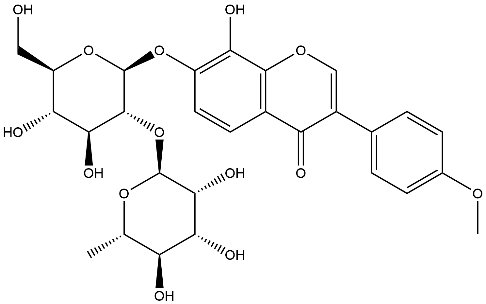 | Retusin-7-O-neohesperidoside | Flavonoids |
| 10 | 8.469 | C15 H10 O6 | 285.0392 | [M-H]^-^ | 285.0394 | -0.491 | 257.00549, 240.96454, 216.948495, 198.91833, 174.90018, 150.83679 | 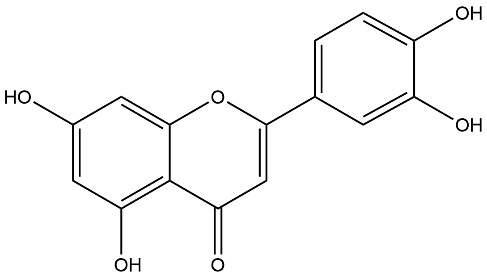 | Luteolin | Flavonoids |
| 11 | 9.580 | C16 H12 O6 | 299.0549 | [M-H]^-^ | 299.0550 | -0.468 | 284.05405, 271.14679, 227.13638, 201.26089, 165.08572 | 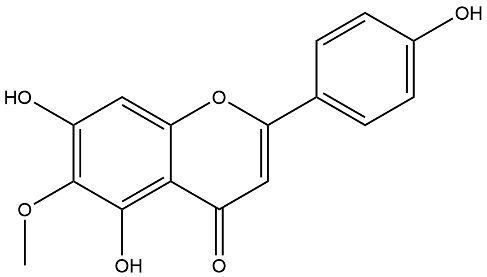 | Hispidulin | Flavonoids |
| 12 | 10.357 | C18 H34 O5 | 329.2316 | [M-H]^-^ | 329.2320 | -1.215 | 311.11176, 293.16467, 229.11169, 210.95567, 170.92079 | 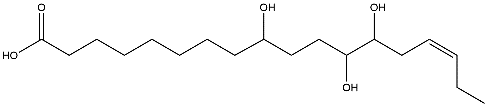 | (15Z)-9,12,13-Trihydroxy-15-octadecenoic acid | Fatty acids |
| 13 | 13.795 | C18 H32 O4 | 311.2213 | [M-H]^-^ | 311.2217 | -1.221 | 293.10272, 275.17099, 253.12469, 235.06638, 223.09506 | 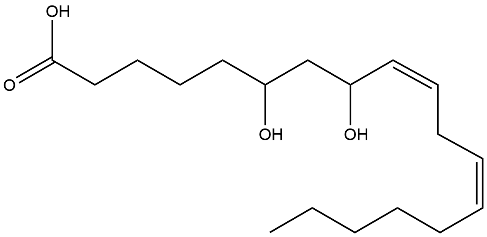 | (9Z,12Z)-6,8-Dihydroxy-9,12-octadecadienoic acid | Fatty acids |
| 14 | 15.484 | C18 H34 O4 | 313.2370 | [M-H]^-^ | 313.2373 | -1.181 | 295.21387, 277.14398, 194.94974, 182.93834 | 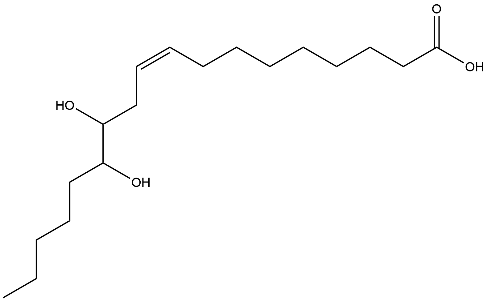 | (+/-)12(13)-DiHOME | Fatty acids |
| 15 | 16.054 | C18 H34 O4 | 313.2369 | [M-H]^-^ | 313.2373 | -1.277 | 295.18872, 277.11288, 251.13235, 200.96495,171.02914 | 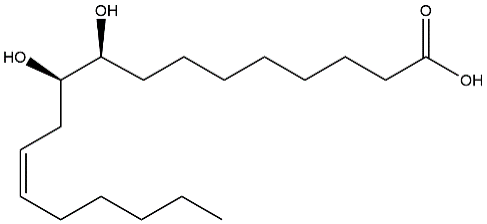 | (+/-)9,10-dihydroxy-12Z-octadecenoic acid | Fatty acids |
| 16 | 26.079 | C16 H32 O2 | 255.2319 | [M-H]^-^ | 255.2320 | -0.392 | 237.14500, 208.77058, 186.80859 | 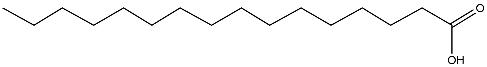 | Palmitic Acid | Fatty acids |
| 17 | 27.227 | C16 H32 O2 | 255.2319 | [M-H]^-^ | 255.2319 | -0.157 | 237.16039, 208.84904, 186.84799, 180.98842 | 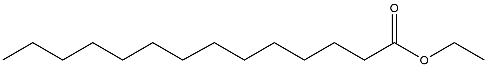 | Ethyl myristate | Esters |
| 18 | 28.662 | C16H32O3 | 271.2268 | [M-H]^-^ | 271.2268 | 0.037 | 253.16396, 225.14417, 205.04897 | 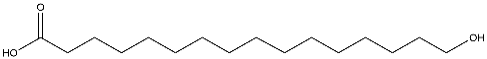 | 16-Hydroxyhexadecanoic acid | Fatty acids |
| 19 | 31.298 | C18H32O2 | 279.2317 | [M-H]^-^ | 279.2319 | -0.716 | 261.21027, 205.13068 | 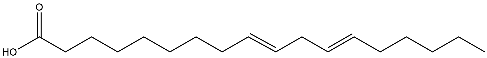 | Linoleic acid | Fatty acids |
| 20 | 37.503 | C24 H40 O4 | 391.2838 | [M-H]^-^ | 391.2843 | -1.150 | 373.21671, 347.27698, 279.36707, 255.18323 | 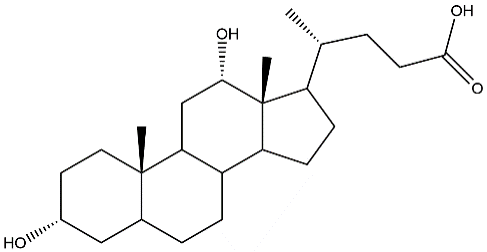 | Deoxycholic acid | Steroids |

**Table S2.** Targets related to 7 identified corn silk components.

| MOLID | Compound | Target |
| --- | --- | --- |
| MOL000842 | sucrose | AR |
| MOL000842 | sucrose | ALAD |
| MOL000842 | sucrose | SI |
| MOL000842 | sucrose | LCT |
| MOL000842 | sucrose | G6PC |
| MOL000842 | sucrose | AMY2A |
| MOL000842 | sucrose | Amy1 |
| MOL000842 | sucrose | CHI3L1 |
| MOL000842 | sucrose | HK1 |
| MOL000842 | sucrose | ACSL1 |
| MOL000842 | sucrose | CYP1A2 |
| MOL000842 | sucrose | COL7A1 |
| MOL000842 | sucrose | COL1A1 |
| MOL000842 | sucrose | NR1I3 |
| MOL000842 | sucrose | SREBF1 |
| MOL000842 | sucrose | GLUL |
| MOL000842 | sucrose | ACSL4 |
| MOL004544 | D-(-)-Quinic acid | PTGS2 |
| MOL004544 | D-(-)-Quinic acid | IGHG1 |
| MOL000415 | rutin | TOP-2 |
| MOL000415 | rutin | RELA |
| MOL000415 | rutin | TNF |
| MOL000415 | rutin | IL6 |
| MOL000415 | rutin | CASP3 |
| MOL000415 | rutin | POR |
| MOL000415 | rutin | SOD1 |
| MOL000415 | rutin | CAT |
| MOL000415 | rutin | IL1B |
| MOL000415 | rutin | CXCL8 |
| MOL000415 | rutin | PRKCB |
| MOL000415 | rutin | ALOX5 |
| MOL000415 | rutin | HMGCR |
| MOL000415 | rutin | HAS2 |
| MOL000415 | rutin | GSTP1 |
| MOL000415 | rutin | DIO1 |
| MOL000415 | rutin | C5AR |
| MOL000415 | rutin | INS |
| MOL000415 | rutin | FCER2 |
| MOL000415 | rutin | TBXA2R |
| MOL000415 | rutin | ITGB2 |
| MOL000006 | Luteolin | PTGS1 |
| MOL000006 | Luteolin | AR |
| MOL000006 | Luteolin | PTGS2 |
| MOL000006 | Luteolin | HSP90 |
| MOL000006 | Luteolin | PRSS1 |
| MOL000006 | Luteolin | NCOA2 |
| MOL000006 | Luteolin | PRKACA |
| MOL000006 | Luteolin | DPP4 |
| MOL000006 | Luteolin | PIK3CG |
| MOL000006 | Luteolin | RELA |
| MOL000006 | Luteolin | EGFR |
| MOL000006 | Luteolin | AKT1 |
| MOL000006 | Luteolin | VEGFA |
| MOL000006 | Luteolin | CCND1 |
| MOL000006 | Luteolin | BCL2L1 |
| MOL000006 | Luteolin | CDKN1A |
| MOL000006 | Luteolin | CASP9 |
| MOL000006 | Luteolin | MMP2 |
| MOL000006 | Luteolin | MMP9 |
| MOL000006 | Luteolin | MAPK1 |
| MOL000006 | Luteolin | IL10 |
| MOL000006 | Luteolin | RB1 |
| MOL000006 | Luteolin | CDK4 |
| MOL000006 | Luteolin | TNF |
| MOL000006 | Luteolin | JUN |
| MOL000006 | Luteolin | IL6 |
| MOL000006 | Luteolin | CASP3 |
| MOL000006 | Luteolin | TP53 |
| MOL000006 | Luteolin | NFKBIA |
| MOL000006 | Luteolin | XDH |
| MOL000006 | Luteolin | TOP1 |
| MOL000006 | Luteolin | MDM2 |
| MOL000006 | Luteolin | APP |
| MOL000006 | Luteolin | MMP1 |
| MOL000006 | Luteolin | PCNA |
| MOL000006 | Luteolin | ERBB2 |
| MOL000006 | Luteolin | PPARG |
| MOL000006 | Luteolin | HMOX1 |
| MOL000006 | Luteolin | CASP7 |
| MOL000006 | Luteolin | ICAM1 |
| MOL000006 | Luteolin | MCL1 |
| MOL000006 | Luteolin | BIRC5 |
| MOL000006 | Luteolin | IL2 |
| MOL000006 | Luteolin | CCNB1 |
| MOL000006 | Luteolin | TYR |
| MOL000006 | Luteolin | IFNG |
| MOL000006 | Luteolin | IL4 |
| MOL000006 | Luteolin | TOP2A |
| MOL000006 | Luteolin | GSTP1 |
| MOL000006 | Luteolin | BIRC4 |
| MOL000006 | Luteolin | SLC2A4 |
| MOL000006 | Luteolin | INSR |
| MOL000006 | Luteolin | CD40LG |
| MOL000006 | Luteolin | PTGES |
| MOL000006 | Luteolin | NUF2 |
| MOL000006 | Luteolin | ADCY2 |
| MOL000006 | Luteolin | MET |
| MOL001735 | Hispidulin | NOS2 |
| MOL001735 | Hispidulin | PTGS1 |
| MOL001735 | Hispidulin | PTGS2 |
| MOL001735 | Hispidulin | DPP4 |
| MOL001735 | Hispidulin | HSP90 |
| MOL001735 | Hispidulin | PIK3CG |
| MOL001735 | Hispidulin | PRKACA |
| MOL001735 | Hispidulin | PRSS1 |
| MOL001735 | Hispidulin | NCOA2 |
| MOL001735 | Hispidulin | NCOA1 |
| MOL001735 | Hispidulin | CALM |
| MOL001735 | Hispidulin | NOS3 |
| MOL001735 | Hispidulin | ACHE |
| MOL001735 | Hispidulin | RHO |
| MOL001735 | Hispidulin | IGHG1 |
| MOL000069 | Palmitic acid | CTSD |
| MOL000069 | Palmitic acid | ADH1B |
| MOL000069 | Palmitic acid | ADH1C |
| MOL000069 | Palmitic acid | PTGS1 |
| MOL000069 | Palmitic acid | PTGS2 |
| MOL000069 | Palmitic acid | RHO |
| MOL000069 | Palmitic acid | IGHG1 |
| MOL000069 | Palmitic acid | NCOA2 |
| MOL000069 | Palmitic acid | BCL2 |
| MOL000069 | Palmitic acid | IL10 |
| MOL000069 | Palmitic acid | TNF |
| MOL000069 | Palmitic acid | COL19A1 |
| MOL000069 | Palmitic acid | GUSBP1 |
| MOL000069 | Palmitic acid | GUSBP1 |
| MOL000069 | Palmitic acid | SLC22A5 |
| MOL000069 | Palmitic acid | PCYT1A |
| MOL008845 | Deoxycholic acid | PGR |
| MOL008845 | Deoxycholic acid | NR3C2 |
| MOL008845 | Deoxycholic acid | NCOA2 |
| MOL008845 | Deoxycholic acid | NCOA1 |
